# Inactivation of the RB1 and PTPN14 tumor suppressors cooperatively enables the carcinogenic activity of the human papillomavirus E7 oncoprotein

**DOI:** 10.64898/2026.03.16.712171

**Published:** 2026-03-17

**Authors:** Pushkal Sinduvadi Ramesh, Angelo A. Nicolaci, Leah E. Graham, Joangela Nouel, Kevin Xu, Jennifer M. Binning, Karl Munger, Elizabeth A. White

**Affiliations:** 1 Department of Developmental, Molecular, and Chemical Biology, Tufts University School of Medicine, Boston, MA, USA; 2 Department of Molecular Oncology, H. Lee Moffitt Cancer Center and Research Institute, Tampa, FL, USA; 3 Cancer Biology PhD Program, University of South Florida, Tampa, FL, USA; 4 Cell and Molecular Biology Graduate Group, University of Pennsylvania Perelman School of Medicine, Philadelphia, PA, USA; 5 Department of Otorhinolaryngology: Head and Neck Surgery, University of Pennsylvania Perelman School of Medicine, Philadelphia, PA, USA; 6 Current address: NYC Public Health Laboratory, New York, NY, USA; 7 Current address: Division of Orthopaedics, Children’s Hospital of Philadelphia, Philadelphia, PA, USA

**Keywords:** Human papillomavirus, E7, tumor suppressor, keratinocyte, immortalization, retinoblastoma protein, PTPN14, Biological Sciences, Microbiology

## Abstract

High-risk human papillomavirus (HPV) E7 proteins bind and inactivate host cellular tumor suppressors and are essential for the immortalization of primary human keratinocytes. E7 proteins from high- and low-risk HPV genotypes bind directly to at least two tumor suppressors, RB1 and PTPN14, and inactivate both. We previously characterized mutations in high-risk HPV E7 proteins that selectively abrogate the ability of E7 to bind either RB1 or PTPN14. Here, we established a genetic complementation system using the E7 mutants defective for binding to RB1 or PTPN14. Neither mutant alone could extend the lifespan of primary keratinocytes. When expressed together, the mutants could, like wild-type high-risk HPV E7, extend keratinocyte lifespan. Both high- and low-risk E7 reduced PTPN14 protein levels and reduced expression of keratinocyte differentiation genes, whereas only high-risk E7 reduced steady-state RB1 levels and induced E2F-dependent genes. Depletion of either RB1 or PTPN14 could cooperate with low-risk HPV6 E7 to extend keratinocyte lifespan, prompting the observation that PTPN14 depletion and RB1 inactivation by HPV E7 acted synergistically to induce certain cell cycle regulatory genes. Our findings advance the model that inactivation of at least two tumor suppressors is required for the carcinogenic activity of high-risk HPV E7. Although RB1 and PTPN14 regulate distinct signaling pathways, their combined inactivation may also contribute to the biological activity of HPV E7.

## Introduction

Human papillomaviruses (HPVs) are double-stranded DNA viruses that infect cutaneous or mucosal epithelial tissues, and several hundred genetically distinct HPV genotypes have been identified (1). Most HPV infections are transient and asymptomatic. However, persistent infection with a subset of high-risk (oncogenic) HPVs can cause mucosal epithelial cancers including cervical, other anogenital, and oropharyngeal cancers (2–4). Low-risk HPVs also infect mucosal tissues but do not cause cancer in healthy individuals at the same anatomical sites.

HPV16 and HPV18 are the most prevalent high-risk HPV genotypes worldwide. They cause most cervical cancers and HPV-associated oropharyngeal cancers in many regions of the world (5). HPV-mediated carcinogenesis is driven by the E6 and E7 oncoproteins, which inactivate cellular tumor suppressor proteins (6). Cellular immortalization is a necessary precursor to carcinogenesis, and the combined expression of high-risk HPV E6 and E7 is necessary and sufficient to immortalize otherwise short-lived primary epithelial cells in culture (7–9). High-risk HPV E7 alone can extend the lifespan of these cells, i.e., enable their growth for several months. Keratinocyte lifespan extension is not a characteristic of low-risk E7 or of high-risk or low-risk E6 proteins (10, 11).

HPV E7 proteins alter cellular signaling by binding to host proteins. Although high- and low-risk HPV E7 proteins share several host cell targets, they have different carcinogenic potential (12, 13). Two of the host cell proteins that are bound by HPV E7 from many different genotypes are the tumor suppressors retinoblastoma protein (RB1) and protein tyrosine phosphatase non-receptor type 14 (PTPN14) (12, 14–16). The crystal structures of the E7-RB1 and E7-PTPN14 interaction sites have been solved, demonstrating that both RB1 and PTPN14 bind directly to E7 (17, 18). E7 binding to either RB1 or PTPN14 requires amino acids in E7 that are highly conserved across the HPV phylogeny (14, 15, 19, 20). Although many other binding partners of E7 have been reported, we are unaware of other E7-host protein interactions that occur via direct binding to similarly highly conserved sites.

HPV E7 proteins bind to RB1 using an LxCxE amino acid sequence in E7 conserved region 2 (CR2) (14, 15). HPV E7 binds predominantly to hypophosphorylated RB1, which is the species that can bind and inhibit E2F transcription factors (21–23). High- and low-risk E7 have different effects on RB1. High-risk HPV E7 proteins bind RB1 with higher affinity than low-risk HPV E7 and more efficiently disrupt the E2F/RB1 complex (14, 24–29). High-risk HPV E7 proteins also target RB1 for degradation, as measured by a decrease in RB1 half-life (30, 31). One consequence of disrupting RB1/E2F complexes is increased transcription of genes required for cell cycle progression from G1 into S phase (32). However, there are differing reports regarding the magnitude of E2F target gene induction by E7. In reporter assays in human, primate, or rodent cell lines, high-risk HPV E7 proteins cause a robust increase in E2F reporter transcription (33–36). In contrast, the effect of low-risk HPV E7 on E2F reporters varies in the same experiments. We are unaware of experiments that have compared the effect of high- vs low-risk HPV E7 on endogenous E2F target genes in human keratinocytes, and consequently the question of whether high- and low-risk HPV E7 have equivalent capacity to induce E2F dependent gene expression remains unresolved.

Like RB1, PTPN14 binds to E7 proteins from many different HPV genotypes (12, 20). Work from several groups has established the mechanism by which HPV E7 proteins bind PTPN14 and target it for degradation. The N-terminus of E7 binds to the ubiquitin ligase UBR4/p600 and the C-terminus binds to PTPN14, forming an E7/PTPN14/UBR4 complex and enabling PTPN14 ubiquitination and degradation (37–39). The structure of the HPV18 E7 C-terminus bound to the C-terminus of PTPN14 has been solved, revealing that HPV18 E7 R84 and L91 make direct contact with PTPN14 via intermolecular hydrogen bonds and hydrophobic interactions, respectively (17). The C-terminal arginine that corresponds to R84 in HPV18 E7 (and R77 in HPV16 E7) is highly conserved (20). Of the 219 HPV genotypes in genus alpha, beta, and gamma, there is an arginine at the same site in 84.5% of the E7 proteins and another basic amino acid (lysine or histidine) at the same site in nearly every other E7. The amino acids in the region of E7 that binds to UBR4 are likewise highly conserved (20).

PTPN14 is a tumor suppressor that negatively regulates the YAP1 oncoprotein, which is a driver of cell stemness and self-renewal (40–46). PTPN14 mutations occur frequently in several cancer types (47, 48). We found that when HPV16 or HPV18 E7 degrades PTPN14, YAP1 becomes active and localizes to the nucleus (49–52). In monolayer cell culture, the primary effect of PTPN14 degradation is decreased expression of early keratinocyte differentiation markers such as keratins 1 and 10 (53). PTPN14 requires YAP1 to regulate differentiation genes (49). Consistent with a reduced commitment to epithelial differentiation, when E7 can degrade PTPN14, keratinocytes are preferentially retained in the basal layer of a stratified epithelium (49). PTPN14 likely lacks phosphatase activity. It does not contain a sequence that, in active protein tyrosine phosphatases, contributes to binding phosphotyrosine, nor does mutation of the putative PTPN14 active site prevent PTPN14 from relocalizing YAP1 to the cytoplasm or restricting cell growth (43, 48, 54). The tumor suppressive capacity of PTPN14 instead depends on its PPxY motifs, which bind to cellular proteins to promote activity of the Hippo pathway upstream of YAP1 (48, 50).

Here, we sought to determine whether and how RB1 inactivation and PTPN14 inactivation cooperate to enable the biological activity of high- and/or low-risk HPV E7. Our experiments address several questions. One question is related to RB1-independent activities of E7. RB1 binding and/or degradation is necessary, but insufficient, for high-risk HPV E7 carcinogenic activity (26, 55–63). Consequently, one or more RB1-independent activities must cooperate with RB1 inactivation to enable transformation by E7, but there is no consensus as to the nature of such an activity. PTPN14 inactivation could be an RB1-independent transforming activity. We and others established that the E7/PTPN14/UBR4 complex can be separated by size-exclusion chromatography from the E7/RB1 complex, that mutation of the RB1, PTPN14, and/or UBR4 binding sites reduces E7 carcinogenic activity, and that many of the genes dysregulated upon PTPN14 inactivation are different from those dysregulated by HPV E7 binding to RB1 (39, 53, 64). However, we lacked genetic evidence in support of two independent activities of HPV E7. A second question is why high- and low-risk HPV E7 proteins share many cellular targets yet only high-risk E7 can transform. Here we addressed both questions. We demonstrate genetic complementation using RB1- and PTPN14-binding deficient mutants of HPV16 or HPV18 E7 in keratinocyte lifespan extension assays. Comparing the effect of high- and low- risk HPV E7 on PTPN14 and RB1, we found that various E7s shared the ability to reduce PTPN14 levels and inhibit differentiation gene expression, but only high-risk E7 caused a robust increase in E2F-dependent gene expression downstream of RB1 inactivation. Unexpectedly, either RB1 knockdown or PTPN14 knockdown cooperated with low-risk HPV E7 in assays of keratinocyte lifespan extension, prompting the finding that PTPN14 knockdown can act synergistically with E7 to promote the expression of certain E2F target genes. We conclude that inactivation of both RB1 and PTPN14 is required for the carcinogenic activity of high-risk HPV E7, and experimentally induced loss of either RB1 or PTPN14 confers carcinogenic properties on low-risk E7.

## Results

### High-risk HPV E7 mutants defective for RB1 or PTPN14 binding complement in *trans* to extend keratinocyte lifespan.

We hypothesized that if RB1 inactivation and PTPN14 inactivation are independent activities of high-risk HPV E7, then a mutant that cannot inactivate RB1 would complement one that cannot inactivate PTPN14 in assays of keratinocyte lifespan extension. To enable genetic complementation experiments, we used pairs of retroviral vectors with complementary selectable markers to deliver two different versions of E7 to the same cell. First, we focused on HPV18 E7. To disrupt binding of E7 to RB1, we used HPV18 E7 ΔDLLC, which harbors a deletion in the LxCxE motif required for E7 to bind RB1 (23, 49). To disrupt binding of E7 to PTPN14, we used HPV18 E7 R84A L91A, which was previously characterized by Yun and colleagues and is altered in two residues that make direct contact with PTPN14 (17). Like HPV18 E7 R84S, which we previously characterized (20), HPV18 E7 R84A L91A was impaired in reducing the steady-state levels of PTPN14 protein but competent to induce E2F target genes PCNA and CCNE2 (Supplemental Figure 1A-B).

To confirm that RB1 and PTPN14 binding defective HPV18 E7 mutants were impaired in keratinocyte lifespan extension assays, we expressed wild-type HPV18 E7, HPV18 E7 ΔDLLC, or HPV18 E7 R84A L91A, or GFP as a negative control, in primary human foreskin keratinocytes (HFKs) (Supplemental Figure 1C-E). HPV18 E7 ΔDLLC (cannot bind RB1) was delivered using a vector conferring resistance to hygromycin whereas HPV18 E7 R84A L91A (cannot bind PTPN14) was delivered using a vector conferring resistance to neomycin. Cells expressing wild-type HPV18 E7 exhibited reduced steady-state levels of RB1 and PTPN14 proteins, increased expression of E2F target genes PCNA and CCNE2, and decreased expression of early differentiation markers KRT1 and KRT10 compared to the GFP control. HPV18 E7 ΔDLLC reduced the steady-state level of PTPN14, but not RB1, and did not induce the expression of PCNA or CCNE2. HPV18 E7 R84A L91A reduced the steady state level of RB1 and induced PCNA and CCNE2 but did not reduce PTPN14 levels. KRT1 and KRT10 expression correlated with the level of PTPN14 in the cells. Neither mutant could promote cell growth comparable to wild-type HPV18 E7 (Supplemental Figure 1F). HPV18 E7 ΔDLLC was unable to extend keratinocyte lifespan (as assessed by time in culture) or promote proliferation (as assessed by cumulative population doublings). A few HPV18 E7 R84A L91A cells were maintained for 50 days, but their proliferative capacity was low throughout the experiment.

**Figure 1:**
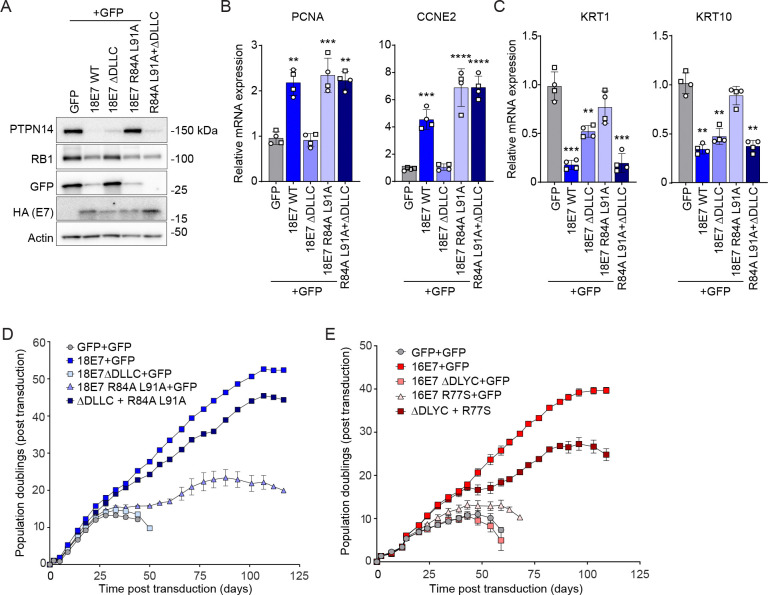
High-risk HPV E7 mutants defective for RB1 or PTPN14 binding complement in *trans* to extend keratinocyte lifespan. (A-D) Primary human keratinocytes were transduced with pairs of retroviruses encoding GFP, wild-type HPV18 E7 (WT), HPV18 E7 ΔDLLC, or HPV18 E7 R84A L91A, with one hygromycin-resistant and one neomycin-resistant retrovirus included in each condition. Each version of HPV18 E7 has an HA epitope tag at its C-terminus. (A) Total cell lysates were subjected to western blotting and probed with antibodies to PTPN14, RB1, GFP, HA, and actin. The expression of (B) E2F target genes (PCNA, CCNE2) and (C) differentiation markers (KRT1, KRT10) were measured by qRT-PCR. Bar graphs represent the mean ± SD of two independent experiments each conducted in duplicate. Statistical significance was determined by one-way ANOVA followed by Holm-Sidak multiple comparison test, comparing each condition to the GFP control (**=*p* ≤ 0.01; ***=*p* ≤ 0.001; ****=*p* ≤ 0.0001). (D) Cell populations were cultured for up to 117 days. Cells were counted at each passage and cell count data was used to calculate cumulative population doublings. Data points indicate the mean and error bars indicate the range for two replicate cell populations. Error bars that are not shown are not visible at this scale. (E) Primary human keratinocytes were transduced with pairs of retroviruses encoding GFP, wild-type HPV16 E7 (WT), HPV16 E7 ΔDLYC, or HPV16 E7 R77S. One hygromycin-resistant and one neomycin-resistant retrovirus was included in each condition. Each version of HPV16 E7 has an HA epitope tag at its C-terminus. Cell populations were cultured for up to 109 days. Cells were counted at each passage and cell count data was used to calculate cumulative population doublings. Data points indicate the mean and error bars indicate the range for two replicate cell populations.

Next, we tested whether the two mutants could complement one another in *trans*. Primary HFKs were transduced with pairs of retroviruses encoding GFP, wild-type HPV18 E7, HPV18 E7 ΔDLLC, or HPV18 E7 R84A L91A, and selected with hygromycin and neomycin. Combined expression of HPV18 E7 ΔDLLC and HPV18 E7 R84A L91A reduced the steady-state levels of RB1 and PTPN14 (Figure 1A), induced the expression of E2F targets PCNA and CCNE2 (Figure 1B), and inhibited the expression of early differentiation markers KRT1 and KRT10 (Figure 1C). In each case, the combined expression of the two mutant proteins recapitulated the effect of wild-type HPV18 E7. In the keratinocyte lifespan extension assay, cells transduced with HPV18 E7 ΔDLLC (cannot bind RB1) doubled only as many times as the GFP control cells (Figure 1D). Cells transduced with HPV18 E7 R84A L91A (cannot bind PTPN14) could be maintained in culture for up to 120 days, but the cells formed colonies poorly and doubled only ~20 times over the three-month assay. In contrast, the combined expression of HPV18 E7 ΔDLLC plus HPV18 E7 R84A L91A enabled HFK growth. The cells transduced with both mutants were, like those expressing wild-type HPV18 E7, readily maintained in culture for up to 120 days. The cells expressing wild-type HPV18 E7 doubled 52 times and the cells expressing both mutants doubled 44 times during the assay.

We repeated the complementation analysis for HPV16 E7, first confirming that delivering the HPV16 E7 mutants with hygromycin or neomycin resistant vectors had the same effects as the puromycin resistant vectors we previously used (20, 53). Like HPV18 E7, wild-type HPV16 E7 induced the expression of E2F targets PCNA and CCNE2 and reduced the expression of early differentiation markers KRT1 and KRT10 (Supplemental Figure 2A, C). HPV16 E7 ΔDLYC (cannot bind RB1) did not induce E2F target expression but impaired the expression of early differentiation markers. HPV16 E7 R77S (cannot bind PTPN14) induced E2F-dependent gene expression but did not inhibit the expression of early differentiation markers (Supplemental Figure 2A, C). In lifespan extension assays lasting 60–70 days, cells transduced with wild-type HPV16 E7 retrovirus doubled 20–25 times, whereas cells transduced with a single retrovirus expressing HPV16 E7 ΔDLYC or HPV16 E7 R77S doubled fewer times and/or could not be maintained in culture for more than 60 days (Supplemental Figure 2B, D). Combined expression of the two defective mutants recapitulated the effect of wild-type HPV16 E7 on PTPN14 and RB1 protein levels and expression of E2F target and early differentiation marker genes (Supplemental Figure 3).

We then tested the ability of the individual HPV16 E7 mutants to complement one another in the lifespan extension assay. Similar to HPV18 E7, cells transduced with one HPV16 E7 mutant retrovirus attained at most ~12 population doublings and stopped proliferating by 75 days (Figure 1E). In contrast, cells transduced with HPV16 E7 ΔDLYC and HPV16 E7 R77S together doubled more than 25 times and, like cells transduced with wild-type HPV16 E7, were readily maintained in culture for approximately four months. Collectively, these findings support that high-risk HPV E7 proteins must bind and degrade both RB1 and PTPN14 in order to extend keratinocyte lifespan. The two activities can be supplied in *trans*, on different molecules of E7.

### High- and low-risk HPV E7 proteins have different effects on RB1 but similar effects on PTPN14.

**Figure 2:**
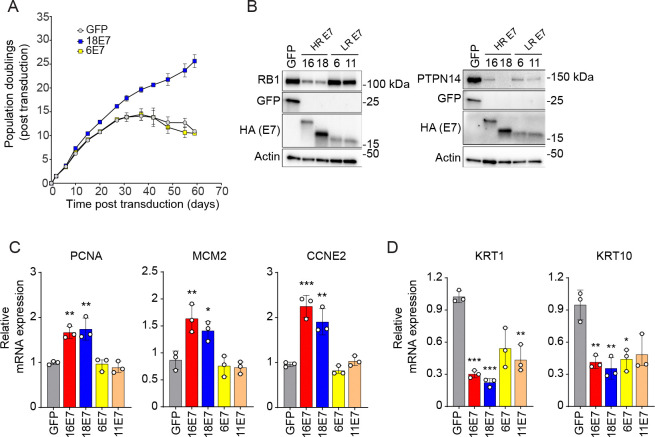
Effect of high-risk and low-risk HPV E7 proteins on RB1 and PTPN14 proteins and target genes. Primary human keratinocytes were transduced with retroviral vectors encoding GFP, HPV16 E7, HPV18 E7, HPV6 E7, or HPV11 E7 and selected with puromycin. Each version of HPV E7 has an HA epitope tag at its C-terminus. (A) Cell pools expressing GFP, HPV18 E7, or HPV6 E7 were cultured for up to 59 days. Cells were counted at each passage and cell count data was used to calculate cumulative population doublings. Data points indicate the mean and error bars indicate the range for two replicate cell populations. (B) Total cell lysates from two experiments were subjected to western blotting and probed with antibodies to PTPN14, RB1, GFP, HA, and actin. HR = high-risk HPV E7; LR = low-risk HPV E7 The expression of (C) E2F targets (PCNA, MCM2, CCNE2) and (D) differentiation markers (KRT1, KRT10) was measured by qRT-PCR. Bar graphs represent the mean ± SD of three replicates. Statistical significance was determined by one-way ANOVA followed by Holm-Sidak multiple comparison test, comparing each condition to the GFP control (*=*p* ≤ 0.05; **=*p* ≤ 0.01; ***=*p* ≤ 0.001).

Both the LxCxE motif and the C-terminal arginine are highly conserved among high- and low-risk HPV E7 proteins. However, high-risk HPV E7 proteins can extend the lifespan of primary HFKs whereas low-risk E7 cannot (11). First, we confirmed that high-risk HPV18 E7 but not low-risk HPV6 E7 could promote proliferation and extend primary keratinocyte lifespan in our assay (Figure 2A). We then compared the effects of two high-risk (HPV16, HPV18) and two low-risk (HPV6, HPV11) E7 on the steady-state levels of RB1 and PTPN14. Consistent with earlier literature (65, 66), high-risk HPV E7 reduced the steady-state levels of RB1 and low-risk E7 did not (Figure 2B). There was a corresponding increase in E2F target gene expression in the cells harboring high-risk, but not low-risk HPV E7 (Figure 2C). In contrast, each HPV E7 reduced the steady-state levels of PTPN14 and the expression of early differentiation markers KRT1 and KRT10 (Figure 2B, D).

### HPV16 E7 and HPV6 E7 share the ability to inhibit differentiation gene expression but not the ability to induce E2F target gene expression.

**Figure 3:**
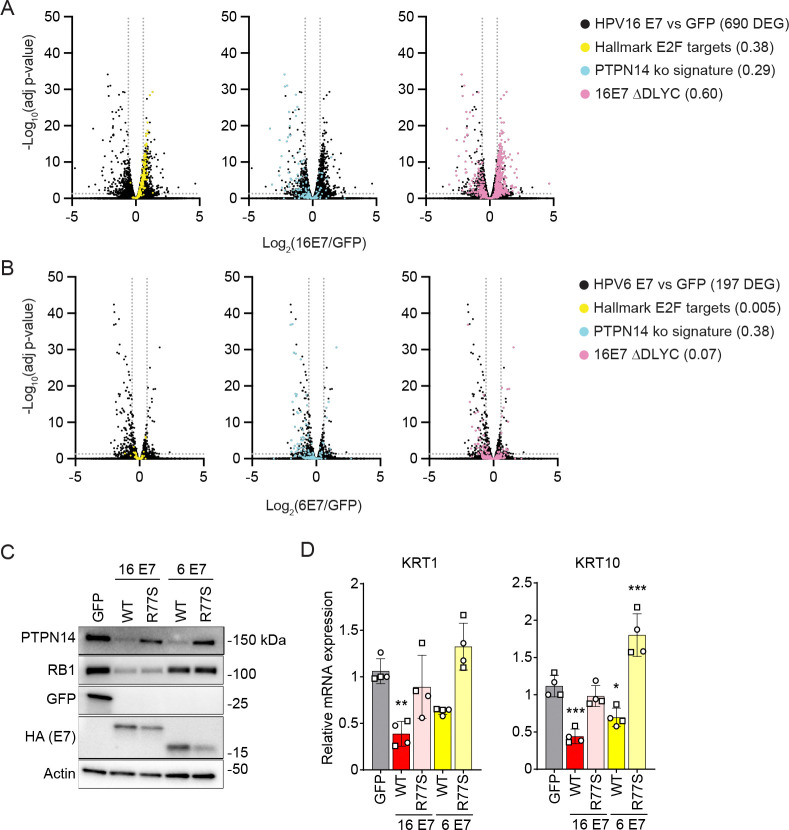
Shared and distinct gene expression patterns in cells expressing high- or low-risk HPV E7. (A, B) Primary HFKs were transduced with retroviral vectors encoding untagged HPV16 or HPV6 E7 proteins or GFP. (A) PolyA selected RNA from HFKs expressing HPV16 E7 or GFP was analyzed by RNA-seq. Each volcano plot shows the gene expression changes in HFK HPV16 E7 vs HFK GFP. 690 genes are differentially expressed with ≥ 1.5 fold change and adjusted *p-*value ≤ 0.05. DEG = differentially expressed genes. (B) PolyA selected RNA from HFKs expressing HPV6 E7 or GFP was analyzed by RNA-seq. Volcano plots show gene expression changes in HFK HPV6 E7 vs HFK GFP. 197 genes are differentially expressed with ≥ 1.5 fold change and adjusted *p-*value ≤ 0.05. For (A) and (B), vertical dashed lines denote 1.5 fold change and horizontal dashed line denotes adjusted *p-*value = 0.05. In the left panel, the 200 genes that comprise the Hallmark E2F targets list are highlighted in yellow. In the center panel, the 140 genes that are differentially expressed upon PTPN14 knockout in HFKs (≥ 1.2 fold change and adjusted *p-*value ≤ 0.05) are highlighted in blue (53). In the right panel, the 713 genes that are differentially expressed in HPV16 E7 cells relative to HPV16 E7 ΔDLYC cells in this experiment are highlighted in pink. The ratios in the figure legends indicate the fraction of genes in a list that were differentially expressed in HFK-E7 vs HFK-GFP cells in this experiment. (C, D) Primary human keratinocytes were transduced with retroviral vectors encoding GFP, wild-type (WT) HPV16 E7, HPV16 E7 R77S, WT HPV6 E7, or HPV6 E7 R77S, and selected with puromycin. Each version of HPV E7 has Flag and HA epitope tags at its C-terminus. (C) Total cell lysates were subjected to western blotting and probed with antibodies to PTPN14, RB1, GFP, HA, and actin. (D) The expression of differentiation markers (KRT1, KRT10) was measured by qRT-PCR. Bar graphs represent the mean ± SD of two independent experiments each conducted in duplicate. Statistical significance was determined by one-way ANOVA followed by the Holm-Sidak multiple comparison test, comparing each condition to the GFP control (*=*p* ≤ 0.05; **=*p* ≤ 0.01; ***=*p* ≤ 0.001).

Next, we sought to more broadly compare the effect of low- vs high-risk HPV E7 on gene expression. We generated HFKs expressing untagged wild-type high-risk HPV16, low-risk HPV6 E7, or an LxCxE deletion mutant form of each one, validated E7 expression by qRT-PCR (Supplemental Figure 4A), and conducted RNA-seq analysis (Figure 3). There were 690 differentially expressed genes (≥ 1.5-fold change and adjusted *p-*value ≤ 0.05) in HFK-16E7 compared to HFK-GFP cells (Figure 3A). The pathways enriched among the upregulated genes included those related to cell division, DNA replication, and DNA repair, a signature that is consistent with HPV16 E7-dependent activation of E2F target genes (Supplemental Figure 4B). The pathways enriched among the downregulated genes included those related to keratinocyte differentiation, which is consistent with HPV16 E7-dependent degradation of PTPN14 and inhibition of epithelial differentiation. We compared the differentially expressed genes to additional gene sets and found that 38% of the hallmark E2F gene list (GSEA gene set M5925, HALLMARK_E2F_TARGETS), 29% of the PTPN14 knockout signature genes defined in (53), and 60% of the genes differentially expressed in the comparison of HPV16 E7 to HPV16 E7 ΔDLYC (Supplemental Figure 5A) were also altered by HPV16 E7 when compared to GFP.

In contrast, only about one third as many genes (197 genes) were differentially expressed in HFK-6E7 vs HFK-GFP (Figure 3B). Like HPV16 E7, low-risk HPV6 E7 reduced the expression of genes associated with epithelial differentiation (Supplemental Figure 4C). HPV16 E7 and HPV6 E7 altered the expression of a similar number of the PTPN14 knockout signature genes: 29% for HPV16 E7 and 38% for HPV6 E7. In contrast to HPV16 E7, HPV6 E7 induced the expression of very few genes, and only a limited number of pathways were represented among those upregulated genes. Only one gene from the hallmark E2F gene list and 7% of the genes differentially expressed in the HPV16 E7 versus the HPV16 E7 ΔDLYC comparison were differentially regulated by HPV6 E7. In addition, very few genes were differentially expressed when we compared HPV6 E7 to HPV6 E7 ΔGLHC (cannot bind RB1) and the changes in E2F target gene expression were minimal (Supplemental Figure 5B). In proliferative cells, HPV6 E7 inhibited differentiation gene expression but did not induce robust E2F target expression.

### HPV6 E7 reduces differentiation gene expression dependent on the C-terminal arginine.

Having observed that HPV16 E7 and HPV6 E7 reduced differentiation gene expression to a comparable degree, we tested whether the C-terminal arginine in HPV6 E7 contributes to differentiation repression. We previously determined that wild-type HPV6 E7 reduces the steady-state levels of PTPN14 but HPV6 E7 R77S does not (20). We transduced primary keratinocytes with retroviruses encoding GFP, HPV16 E7, HPV16 E7 R77S, HPV6 E7, or HPV6 E7 R77S. Steady-state levels of PTPN14 protein were reduced in cells expressing wild-type but not R77S mutants of each E7 (Figure 3C). KRT1 and KRT10 transcript levels were lower in cells expressing the wild-type form of each E7 compared to the corresponding R77S mutant (Figure 3D). Inhibition of differentiation gene expression by either high- or low-risk E7 depends on the conserved C-terminal arginine.

### HPV18 E7 binds to PTPN14 with a higher affinity than HPV16, 6, or 11 E7.

**Figure 4:**
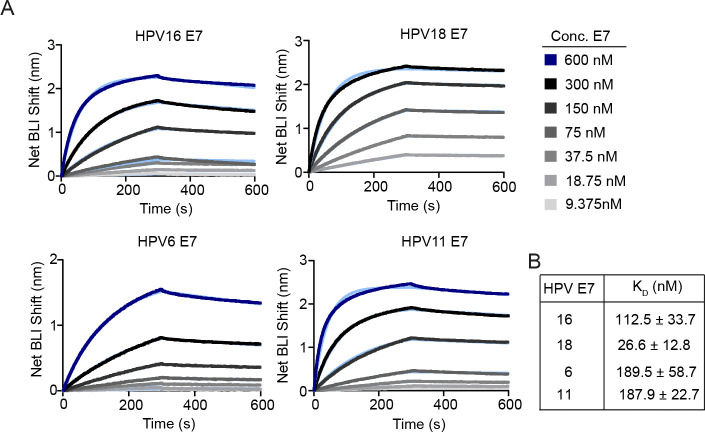
BLI analysis of PTPN14 binding to high- or low-risk HPV E7 proteins. (A) Affinity measurements of twin-strep tagged phosphatase domain of PTPN14 (amino acids 886–1147) against MBP-HPV E7 proteins from four genotypes were conducted using bio-layer interferometry (BLI). Dark blue and black traces represent the raw data; the kinetic fit is shown in light blue underneath. Sensograms shown are representative plots of three replicates for each E7. (B) Table displays equilibrium constants (K_D_) for each interaction.

Having observed that high- and low-risk HPV E7 share the ability to reduce PTPN14 levels and differentiation gene expression, we sought to determine the affinity of the same E7 proteins for the C-terminal phosphatase domain of PTPN14. Full-length HPV16, 18, 6, or 11 E7 were purified and used in biolayer interferometry (BLI) experiments together with the purified C-terminal phosphatase domain of PTPN14 (Figure 4A, Supplemental Figure 6). The dissociation constants were determined for each pairwise interaction between an E7 and PTPN14 (Figure 4B). The affinity of E7 for PTPN14 was highest for HPV18 E7 (*K*_D_ = 26.6 ± 12.8 nM), next highest for HPV16 E7 (*K*_D_ = 112.5 ± 33.7), and lower for the two low-risk HPV6 and HPV11 E7 (*K*_D_ = 189.5 ± 58.7, and 187.9 ± 22.7 nM, respectively) (Figure 4B).

### HPV6 E7 and RB1 knockdown cooperate to extend keratinocyte lifespan.

**Figure 5:**
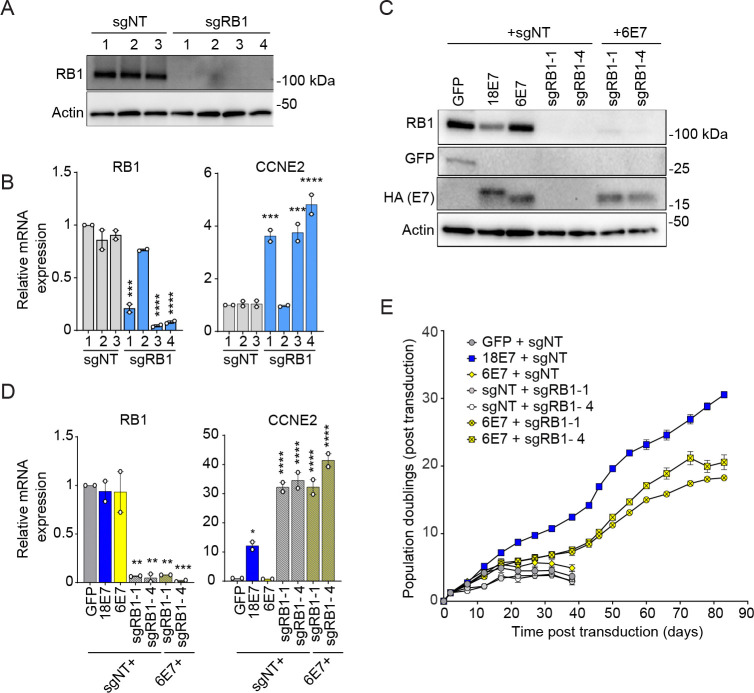
RB1 knockdown promotes lifespan extension in primary keratinocytes expressing HPV6 E7. (A, B) Primary human keratinocytes were transduced with a CRISPRi lentiviral vector encoding dCas9-KRAB MeCP2 and a non-targeting sgRNA or a sgRNA targeting RB1. (A) Total cell lysates were subjected to western blotting and probed with antibodies to RB1 and actin. (B) The expression of RB1 and CCNE2 was measured by qRT-PCR. Bar graphs represent the mean ± range of two replicate cell populations. (C-E) Primary human keratinocytes were transduced with a pair of vectors: one retrovirus encoding GFP, HPV18 E7, or HPV6 E7, plus one CRISPRi lentivirus, and selected with puromycin and neomycin. (C) Total cell lysates were subjected to western blotting and probed with antibodies to RB1, GFP, HA, and actin. (D) The expression of RB1 and CCNE2 was measured by qRT-PCR. Bar graphs represent the mean ± range of two replicate experiments. Statistical significance was determined by one-way ANOVA followed by the Holm-Sidak multiple comparison test, comparing each condition to the GFP control (*=*p* ≤ 0.05; **=*p* ≤ 0.01; ***=*p* ≤ 0.001; ****=*p* ≤ 0.0001). (E) Cell populations were cultured for up to 83 days. Cells were counted at each passage and cell count data was used to calculate cumulative population doublings. Data points indicate the mean and error bars indicate the range for two replicate cell populations.

Having observed that the magnitude of RB1 inactivation distinguishes high-risk from low-risk E7, we hypothesized that knockdown of RB1 would complement a low-risk HPV E7 in a keratinocyte lifespan extension assay. We generated all-in-one CRISPR interference (CRISPRi) lentiviruses to reduce RB1 transcript levels in primary HFKs. The CRISPRi vector encodes dCas9-KRAB-MeCP2 (67) plus one of three non-targeting (sgNT) sgRNAs or one of four sgRNAs directed to the RB1 promoter. Three of the RB1 CRISPRi sgRNAs reduced RB1 protein and RNA levels and induced expression of the E2F target CCNE2 (Figure 5A, B). We transduced primary HFKs with a pair of vectors: the CRISPRi sgNT or sgRB1 vector plus a second vector encoding GFP, high-risk HPV18 E7, or low-risk HPV6 E7. RB1 depletion reduced RB1 protein and RNA levels and induced CCNE2 in the presence or absence of HPV6 E7 (Figure 5C, D). Neither HPV6 E7 nor RB1 depletion alone extended the lifespan of HFKs compared to the GFP control (Figure 5E). In contrast, combined expression of HPV6 E7 and depletion of RB1 enabled long-term propagation of the cells. Like the HPV18 E7 cells, the HPV6 E7 + CRISPRi sgRB1 cells were readily maintained in culture for 90 days, and they doubled up to 20 times during the experiment.

### HPV6 E7 and PTPN14 knockdown cooperate to extend keratinocyte lifespan.

**Figure 6:**
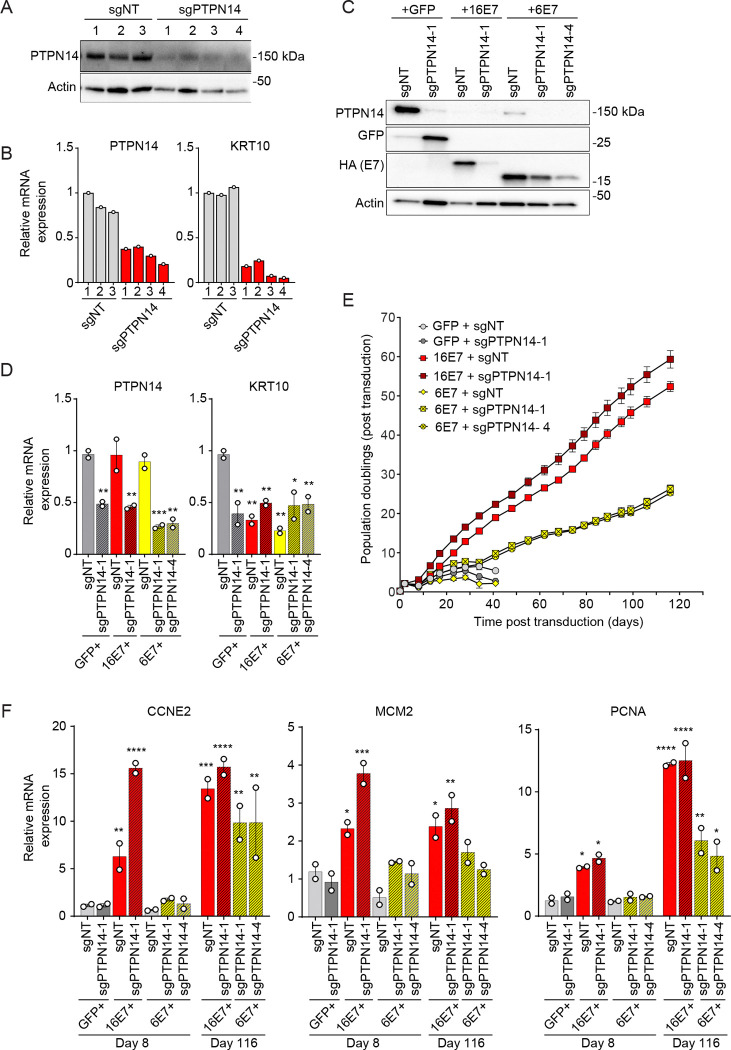
PTPN14 knockdown promotes lifespan extension in primary keratinocytes expressing HPV6 E7. (A, B) Primary human keratinocytes were transduced with a lentiviral CRISPRi vector encoding dCas9-KRAB MeCP2 and a non-targeting sgRNA or a sgRNA targeting PTPN14. (A) Total cell lysates were subjected to western blotting and probed with antibodies to PTPN14 and actin. (B) The expression of PTPN14 and KRT10 was measured by qRT-PCR. (C-E) Primary human keratinocytes were transduced with a combination of one retrovirus encoding GFP, HPV16 E7, or HPV6 E7, plus one CRISPRi lentivirus and selected with puromycin and neomycin. (C) Cell lysates were subjected to western blotting and probed with antibodies to PTPN14, GFP, HA, and actin. (D) The expression of PTPN14 and KRT10 was measured by qRT-PCR. Bar graphs represent the mean ± range of two replicate cell populations. Statistical significance was determined by one-way ANOVA followed by the Holm-Sidak multiple comparison test, comparing each condition to the GFP control (*=*p* ≤ 0.05; **=*p* ≤ 0.01). (E) Cell populations were cultured for up to 106 days. Cells were counted at each passage and cell count data was used to calculate cumulative population doublings. Data points indicate the mean and error bars indicate the range for two replicate cell populations. (F) Day 8 and day 116 RNA was analyzed from cell populations in (E). The expression of CCNE2, MCM2 and PCNA was measured by qRT-PCR. Bar graphs represent the mean ± range of two replicate cell populations. Statistical significance was determined by one-way ANOVA followed by Holm-Sidak multiple comparison test, comparing each condition to the day 8 GFP + sgNT control (*=p ≤ 0.05; **=p ≤ 0.01; ***=p ≤ 0.001; ****=p ≤ 0.0001).

Considering that both high- and low risk HPV E7 reduce the steady-state levels of PTPN14, albeit to varying degrees, we predicted that depletion of PTPN14 would not complement a low-risk HPV E7 in a keratinocyte lifespan extension assay. We generated vectors for CRISPRi depletion of PTPN14 and confirmed that they reduced PTPN14 protein and RNA levels and expression of the differentiation marker KRT10 (Figure 6A, B). We transduced primary HFKs with one vector that expressed GFP control, high-risk HPV16 E7, or low-risk HPV6 E7, plus a second CRISPRi sgNT or sgPTPN14 vector (Figure 6C, D). PTPN14 knockdown reduced PTPN14 transcript levels but did not further reduce KRT10 expression in HPV16 E7 or HPV6 E7 cells. Neither HPV6 E7 nor PTPN14 depletion alone extended keratinocyte lifespan compared to control cells (Figure 6E). In contrast, PTPN14 knockdown cooperated with HPV6 E7 to extend keratinocyte lifespan, and the cells transduced with both vectors were readily maintained in culture for four months. Depleting PTPN14 also increased the growth of HPV16 E7 cells, enabling HPV16 E7 + sgPTPN14 cells to double about five more times than HPV16 E7 + sgNT cells during the four-month assay.

### PTPN14 knockdown cooperates with high-risk HPV E7 to increase the expression of E2F target genes.

Given the unexpected finding that CRISPRi knockdown of PTPN14 increased the proliferative capacity (represented by population doublings) of cells expressing HPV16 E7 and cooperated with low-risk HPV E7 to extend keratinocyte lifespan, we assessed gene expression in the same cells. YAP1 and E2F can cooperate to promote the expression of some cell cycle genes (68–73). We found that at the early time point post transduction (day 8), CCNE2 mRNA levels were induced by HPV16 E7, but were higher in HPV16 E7 + sgPTPN14 cells than in HPV16 E7 + sgNT, GFP + sgNT, or any HPV6 E7 cells (Figure 6F). At the end of the experiment (day 116), CCNE2 levels in HPV16 E7 + sgPTPN14 cells were similar to those detected at the early time point and had increased to a comparable level in HPV16 E7 + sgNT cells. CCNE2 transcript levels in HPV6 E7 + sgPTPN14 cells increased and were higher in those cells on day 116 than in HPV16 E7 + sgNT cells on day 8. Other cell cycle genes exhibited slightly different expression patterns. There was an early synergistic effect of HPV16 E7 and sgPTPN14 on MCM2, but MCM2 levels did not increase over time in the HPV6 E7 + sgPTPN14 cells. There was not a synergistic effect of HPV16 E7 and sgPTPN14 on PCNA, but HPV6 E7 + sgPTPN14 had higher PCNA levels on day 116 than at day 8 (Figure 6F). E7 and sgPTPN14 did not act synergistically to reduce KRT10 levels (Supplemental Figure 7).

**Figure 7:**
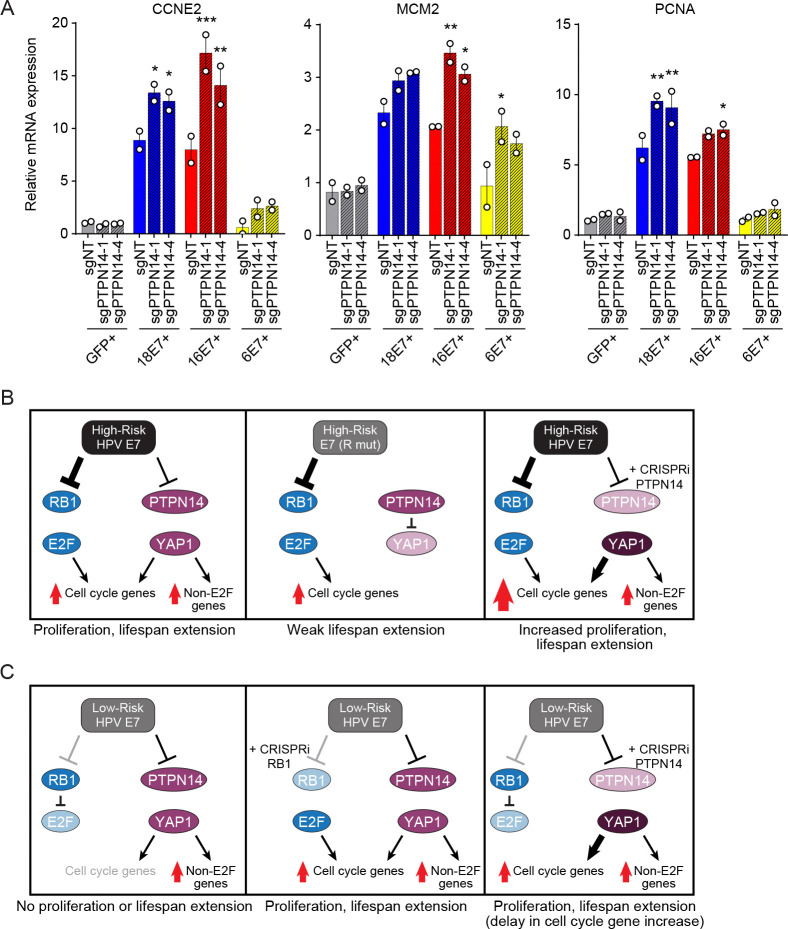
PTPN14 knockdown cooperates with HPV E7 to increase the expression of cell cycle genes. (A) Primary human keratinocytes were transduced with a retrovirus encoding GFP, HPV16 E7, HPV18 E7, or HPV6 E7 plus a CRISPRi lentivirus and selected with puromycin and neomycin. The expression of CCNE2, MCM2, and PCNA was measured by qRT-PCR. Bar graphs represent the mean ± range of two replicate cell populations. Statistical significance was determined by one-way ANOVA followed by Holm-Sidak multiple comparison test, comparing each condition to the GFP + sgNT control (*=*p* ≤ 0.05; **=*p* ≤ 0.01; ***=*p* ≤ 0.001). (B) Proposed model that high-risk HPV E7s degrade RB1 and PTPN14, activating both cell cycle gene expression and an E2F-independent gene expression program downstream of YAP1 (left panel). In the absence of PTPN14 degradation, cell cycle gene induction is robust but insufficient for lifespan extension (center panel). PTPN14 depletion increases the expression of cell cycle genes (and perhaps other YAP1 target genes) in high-risk HPV E7 cells (right panel). (C) Low-risk HPV E7s degrade PTPN14, but not RB1, and cannot extend keratinocyte lifespan (left panel). RB1 depletion increases cell cycle gene expression and cooperates with low-risk E7 to extend keratinocyte lifespan (center panel). PTPN14 depletion increases cell cycle and other YAP1 target gene expression and cooperates with low-risk E7 to extend keratinocyte lifespan (right panel).

We repeated the transductions and expanded the conditions to include two high-risk HPV E7s and two CRISPRi constructs targeting PTPN14 (Supplemental Figure 8A, B). Twenty-six days post transduction, there was a synergistic effect of either HPV16 or HPV18 E7 + sgPTPN14 on CCNE2 levels (Figure 7A). MCM2 and PCNA levels followed a similar pattern although the magnitude of additional induction by sgPTPN14 varied. The expression of the three genes was lower in HPV6 E7 cells than in the high-risk E7 cells, but even at the 26 day time point some synergy between HPV6 E7 and sgPTPN14 began to emerge. There was no additional reduction in KRT10 levels when we combined E7 and PTPN14 knockdown (Supplemental Figure 8C). Overall, at early times post transduction, there was a synergistic effect of high-risk HPV16 E7 and PTPN14 knockdown on cell cycle genes that we did not observe for HPV6 E7. With additional passage, the expression of cell cycle genes increased in high- or low-risk HPV E7 + sgPTPN14 cells, and those cells could be maintained in culture for several months. Of the cell cycle genes that we tested, the combined effect of HPV E7 + PTPN14 knockdown on gene expression was strongest for CCNE2.

## Discussion

Our previous results prompted the hypothesis that RB1 inactivation and PTPN14 inactivation are separate activities of high-risk HPV E7 proteins that are both required for E7 carcinogenic activity. Here, we tested this hypothesis using mutants of high-risk HPV E7 in genetic complementation experiments. When we co-expressed mutants of HPV16 or HPV18 E7, providing the LxCxE motif in one E7 molecule and the PTPN14 binding site in another, the combination was sufficient to recapitulate the effect of wild-type high-risk HPV16 or HPV18 E7 in several assays. In particular, two individually impaired mutants cooperated to extend the lifespan of primary keratinocytes (Figure 1). We note that compared to wild-type E7, combined expression of the two mutants enabled cell propagation for the same length of time but supported somewhat fewer population doublings (Figure 1). The difference could be related to the E7 dimerization activity (74). Heterodimers consisting of one LxCxE mutant and one R mutant E7 may form in the complemented cells, perhaps inactivating RB1 and/or PTPN14 less effectively than wild-type E7 homodimers. Regardless, cells expressing either wild-type E7 or the two mutants together exhibited both lifespan extension (reflected by time in culture) and induction of proliferation (reflected by population doublings), the combination of which was not exhibited by cells expressing either mutant alone. The ability of HPV E7 to extend keratinocyte lifespan over 3–4 months is well correlated with its ability to immortalize keratinocytes in combination with HPV E6 (11), and keratinocyte immortalization is an essential early step in HPV carcinogenesis. Our results support that RB1 inactivation and PTPN14 inactivation are independent activities that are both required for keratinocyte immortalization.

High- and low-risk HPV E7 proteins engage several of the same cellular targets but have different biological activities. We sought to determine whether and how different effects on RB1 and/or PTPN14 account for the differences between high- and low-risk HPV E7 proteins. Consistent with previous publications (65, 66), high-risk but not low-risk E7 reduced the steady-state levels of RB1 protein (Figure 2). Earlier studies demonstrated that low-risk E7 can bind to RB1, disrupt the interaction of E2F with RB1, and induce the expression of E2F reporter constructs to varying degrees, but they did not test the effect of low-risk E7 on endogenous E2F target genes (33–36). Here we found that E2F target gene expression was inversely related to the level of RB1 protein in HPV E7-expressing cells. High-risk HPV E7 reduced RB1 levels and caused robust activation of E2F-dependent gene expression whereas low-risk E7 had little effect on RB1 levels or E2F-dependent gene expression (Figure 2, 3). In contrast, both high-and low-risk HPV E7 reduced the steady-state levels of PTPN14, albeit to varying degrees (Figure 2). High- and low-risk E7 expression reduced the expression of epithelial differentiation markers, in both cases dependent on the C-terminal arginine that binds PTPN14 (Figure 3).

We predict that the E7/RB1 and E7/PTPN14 interactions are both important for HPV persistent infection in stratified epithelia. Here, we cultured cells in conditions that support robust proliferation, much like the cells in the proliferative basal layer of a stratified epithelium. A possible explanation for our finding that low-risk HPV E7 had a minimal effect on E2F target gene expression is that in proliferative cells, the baseline level of E2F target gene expression is relatively high and is not increased by low-risk E7. It is possible that in postmitotic cells low-risk HPV E7 can induce E2F target expression. Indeed some, but not all, experiments in tissue models show that low-risk HPV E7 induces expression of the E2F target *PCNA* in suprabasal, differentiated keratinocytes (10, 75). In contrast, both high- and low-risk HPV E7 proteins reduced PTPN14 levels and differentiation gene expression. The observation that both high-risk and low-risk E7 proteins reduce PTPN14 levels suggests that inactivation of PTPN14 is a conserved E7 function likely important for the HPV life cycle. In other work we determined that when high-risk HPV E7 proteins degrade PTPN14, they activate YAP1, thereby inhibiting differentiation gene expression and promoting the retention of keratinocytes in the basal epithelial compartment (49). More experiments will be required to determine whether low-risk HPV E7 proteins similarly activate YAP1 to promote basal epithelial identity.

To determine why high-risk E7 can immortalize keratinocytes but low-risk E7 cannot, we modified the complementation approach, testing whether depletion of RB1 or depletion of PTPN14 could cooperate with low-risk HPV6 E7 to extend the lifespan of primary keratinocytes. The observation that RB1 depletion plus HPV6 E7 cooperated to extend keratinocyte lifespan is likely related to the increased expression of E2F targets in 6E7 + sgRB1 cells compared to 6E7 plus sgNT cells (Figure 5). However, given that high- and low-risk E7 differed largely in their effects on RB1, it was unexpected that PTPN14 depletion also cooperated with HPV6 E7 to extend keratinocyte lifespan (Figure 6). At early times post-transduction, there was a synergistic effect of high-risk E7 and sgPTPN14 on cell cycle genes, especially CCNE2. Over time, low-risk HPV6 E7 plus sgPTPN14 could be maintained in culture and the CCNE2 levels in those cells increased.

To better understand how PTPN14 knockdown could cooperate with HPV6 E7 to extend keratinocyte lifespan, we considered the model, reported in the literature, that certain cell cycle control genes, including CCNE2, are subject to cooperative regulation by E2F and YAP1 (Figure 7B) (68–73). Our findings are consistent with such cooperative regulation. We anticipate that the induction of robust proliferation and lifespan extension by HPV E7 requires an increase in cell cycle gene expression past a certain threshold. RB1 inactivation alone is insufficient to extend keratinocyte lifespan (Figure 5), meaning that there is likely a second, E2F-independent, contribution of YAP1. We propose that high-risk HPV E7s degrade RB1 and PTPN14 and drive both the E2F-dependent cell cycle gene expression program and YAP1-dependent gene expression (Figure 7B, left). Several of our other findings are consistent with this model. C-terminal arginine mutants of high-risk HPV E7 scored poorly or not at all in the lifespan extension assay (Figure 7B, center). PTPN14 knockdown in cells expressing high-risk E7 increases the level of certain cell cycle genes past that of high-risk E7 alone (Figure 7B, right).

Low-risk HPV E7 have modest effects on RB1/E2F in proliferative cells and do not extend keratinocyte lifespan (Figure 7C, left). In lifespan extension assays low-risk HPV E7 cooperated with RB1 knockdown, which likely increases effects on cell cycle genes through a direct effect on E2F (Figure 7C, center), and with PTPN14 knockdown, which likely increases the YAP1-dependent contribution to cell cycle gene expression (Figure 7C, right). It should be noted that PTPN14 knockdown alone did not extend keratinocyte lifespan (Figure 6E), meaning that either the modest effect of low-risk E7 on E2F genes or another activity of low-risk E7 also contributes in the lifespan extension assay.

Several of our observations suggest that the contribution of YAP1 to cell cycle gene expression may initially occur in a small number of cells or at a low level. For example, if in HPV6 E7 plus sgPTPN14 cells there are a few cells with elevated CCNE2, selective pressure resulting in the expansion of those cells could enable the outgrowth of a population and the apparent increase in CCNE2 levels over time. Future experiments will aim to determine which genes are regulated in such a manner and how their regulation occurs. The experiments with C-terminal arginine mutants of high-risk HPV E7 highlight that YAP1 activation is required for long-term propagation of high-risk HPV E7 cells. YAP1 could contribute via cooperative effects on cell cycle genes, effects on E2F-independent YAP1 targets, or both.

Our model could help to explain several findings in the human cancer biology literature. Germline PTPN14 mutations predispose individuals to develop basal cell carcinoma and cervical cancer (51), and our data help to explain how genetic inactivation of PTPN14 could increase carcinogenic potential both in the presence and absence of HPV infection. In addition, there are recent reports that certain HPV genotypes previously classified as low-risk can be carcinogenic at specific anatomical sites (76), but the level of PTPN14 expression in cells in which those cancers arise is not known. It is possible that low-risk HPV E7 has some carcinogenic potential in cells that do not express PTPN14. In the long term, it will be important to determine how PTPN14 inactivation and RB1 inactivation synergize to regulate gene expression in experimental models and to contribute to cancer development *in vivo*.

Overall, much of the biological activity of high- and low-risk HPV E7 proteins depends on inactivation of RB1 and PTPN14. The two tumor suppressors regulate separate pathways and have both distinct and synergistic effects on cellular gene expression. The degree to which E7 proteins inactivate RB1 vs PTPN14 appears to account for much of the difference between high- and low-risk E7 proteins. One long-term goal is to better understand how the balanced regulation of RB1 and PTPN14 controls gene expression. We note that many binding partners of E7 besides RB1 and PTPN14 have been reported (13). A second important long-term goal is to determine whether additional host cell targets are required for the carcinogenic activity of certain HPV E7 proteins, for their ability to promote HPV persistent infection, or both.

## Materials and methods

### Plasmids and cloning

#### HPV E7 expression in human keratinocytes.

MSCV-puro C-terminal FlagHA retroviral expression vectors encoding HPV16 E7 wild-type (WT), HPV16 E7 ΔDLYC, HPV16 R77S, HPV18 E7 WT, HPV18 E7 R84S, HPV6 E7 or GFP vector controls were previously described (20, 39) (Supplemental Table 1). Additional vectors encoding various HPV E7 were cloned by Gateway recombination of pDONR E7 plasmids with MSCV-P C Flag HA GAW, MSCV-neo-C-HA GAW or MSCV-hygro C-HA GAW destination vectors. The R84A L91A mutation was introduced by site-directed mutagenesis into pDONR 18E7-Kozak and recombined into destination vectors with neomycin selection as previously described.

#### Biolayer interferometry.

All constructs were generated by standard PCR and isothermal assembly cloning methods unless otherwise noted. gBlocks encoding HPV18, HPV16, HPV11, HPV6 E7, and the C-terminal phosphatase domain of PTPN14 (residues 886–1147) were purchased from Integrated DNA Technologies. E7 constructs were cloned into a modified pET15b vector containing an N-terminal MBP-tag, His-tag, and tobacco etch virus (TEV) cleavage site (MHT-tag). PTPN14 was cloned into the N-terminal MHT-tagged pET15b vector with an additional C-terminal Twin-Strep (TS) tag. Constructs were verified by Sanger sequencing (Genewiz/Azenta Life Sciences).

#### CRISPRi.

The KRAB-dCas9-MeCP2 cassette was excised from lentiCRISPRi vector (Addgene #170068) by double digestion with BamHI and AgeI and gel purified. The cassette was ligated into the corresponding BamHI/AgeI sites of plentiCRISPRv2 backbones carrying neomycin (Addgene #98292) or puromycin (Addgene #52961) selection markers to create lentiCRISPRi_Neo and lentiCRISPRi_Puro. The constructs were propagated in Stbl3 E. coli, purified by midi-prep, and verified by sequencing. Sequences for three distinct non-targeting sgRNAs were previously reported (77) and four independent sgRNAs targeting RB1 or PTPN14 promoters were chosen using CRISPick (78). The sgRNA sequences were phosphorylated and annealed, and the resulting double-stranded inserts were ligated into Esp3I-digested lentiCRISPRi_Neo or lentiCRISPRi_Puro vectors.

### Cell culture, virus production, and lifespan extension assay

Deidentified primary human foreskin keratinocytes (HFKs) were obtained from the Skin Biology and Diseases Resource-based Center (SBDRC) at the University of Pennsylvania. Cells were cultured in Keratinocyte serum free media (K-SFM, ThermoFisher) modified as previously described (12) and all experiments were performed starting with HFK at passage 4 after initial isolation. Retroviral and lentiviral particles were generated by transient transfection of HEK293T cells as previously described (12). HPV E7 expressing cell populations were established by transduction with MSCV retroviral vectors, followed by appropriate selection. RB1 and PTPN14 knockdown or nontargeting control primary HFKs were established by transduction with lentiCRISPRi vectors, followed by appropriate selection. HPV E7 expression and CRISPRi in combination was achieved by simultaneous transduction of MSCV retroviral vectors plus a lentiviral vector and combined selection with neomycin plus puromycin. Following transduction, cells were trypsinized, counted, and seeded in 10 cm dishes for selection with appropriate antibiotic for 3–5 days to establish stable populations. Once selected, cells were maintained in K-SFM and passaged every 4–5 days for up to four months. At each passage cells were counted and cell count data was used to calculate cumulative population doublings (PD). Population doublings for a single passage were calculated as PD = 3.32 (log(number of cells harvested / number of cells seeded)). Each experiment consisted of a minimum of two independent replicates conducted simultaneously unless otherwise specified.

### Western blotting

Whole cell lysates were prepared using RIPA buffer supplemented with protease and phosphatase inhibitors. The samples were sonicated to obtain homogeneous lysates. Protein concentrations were quantified using the Bradford assay. Equal amounts of protein were resolved using Mini-Protean or Criterion Tris/Glycine SDS-PAGE gels (Bio-Rad) and transferred to polyvinylidene difluoride (PVDF) membrane. Membranes were blocked with 5% non-fat dry milk in TBST (Tris buffered saline of pH 7.4 with 0.05% Tween 20) and probed with primary and HRP-conjugated secondary antibodies (Supplemental Table 2). Signals were detected by chemiluminescence.

### RNA extraction and qRT-PCR

Total cellular RNA was isolated using the NucleoSpin RNA extraction kit (Macherey-Nagel/Takara). cDNA was generated using the high-capacity cDNA reverse transcription kit (Applied Biosystems). The synthesized cDNAs were used as a template for qRT-PCR using Fast SYBR green master mix (Applied Biosystems) and a QuantStudio 3 system (ThermoFisher Scientific). KiCqStart SYBR green primers for qRT-PCR (Millipore Sigma) were used to quantify the following transcripts: PCNA, CCNE2, MCM2, RB1, KRT1, KRT10, PTPN14, and GAPDH. All qRT-PCR data were normalized to GAPDH using the delta-delta Ct method (79).

### RNA-seq

Primary HFKs were transduced with retroviruses encoding GFP, untagged HPV16 E7, untagged HPV16 E7 ΔDLYC, untagged HPV6 E7, or untagged HPV6 E7 ΔGLHC. Each transduction was performed in triplicate, and independent replicates were maintained at each subsequent step. Total RNA was isolated using the RNeasy mini kit (Qiagen). PolyA selection, reverse transcription, library construction, sequencing, and initial analysis were performed by Novogene. Differentially expressed genes were selected based on a 1.5-fold change and adjusted *p-* value ≤0.05. Biological pathways for which genes are enriched among the differentially expressed genes were identified using DAVID (80, 81). GO terms in enrichment analyses are displayed in rank order by adjusted p-value. RNA-seq data have been deposited in NCBI GEO with accession number GSE319393.

### Protein expression and purification

For E. *coli* expression, plasmids were transformed into E. *coli* BL21(DE3) (Invitrogen) cells, grown in Luria-Bertani (LB) medium at 37°C to an optical density of 0. 6–0.8, and induced with 0.5 mM iPTG overnight at 18°C. Cells were harvested at 5000 × *g*, lysed, and clarified at 40,000 × *g* in lysis buffer containing 20 mM Tris pH 7.5, 500 mM NaCl, 25 mM Imidazole, and 5 mM beta-mercaptoethanol (BME). All proteins were initially subjected to Ni-NTA affinity purification. Clarified lysates were applied to the resin and eluted with a linear gradient of imidazole (25 mM - 500 mM imidazole in 20 mM Tris pH 8.0, 150 mM NaCl, 5 mM BME).

#### HPV E7.

Following Ni-NTA affinity purification, MHT-tagged E7 constructs were purified by anion-exchange chromatography using a Q HP column (Cytiva, 17115401). E7 was eluted from the ion-exchange column with a linear gradient of NaCl (25 mM - 1000 mM NaCl in 20 mM Tris pH 8.0, 5 mM BME. E7 was concentrated using a 30 kDa molecular weight cutoff (MWCO) Amicon centrifugal filter (Millipore) then size exclusion chromatography (SEC) on Superdex 200 (Cytiva, SD200) was used as the final purification step. The final concentration of each E7 construct was determined by measuring absorbance at 280nm and E7 was stored in 20 mM HEPES, pH 7.5, 150 mM NaCl, 1 mM TCEP at −80°C for all subsequent experiments.

#### Twin-strep-tagged PTPN14 phosphatase domain.

Following Ni-NTA affinity purification, MHT-PTPN14-TS was purified by a second round of affinity purification using a MBPTrap HP (Cytiva) column and elution buffer containing 20 mM Tris, pH 7.5, 300 mM NaCl, 1% Maltose, 5 mM BME. The MHT-tag was removed with recombinant tobacco etch virus (rTEV) protease. TEV-treated samples were subjected to another round of Ni-NTA affinity purification (HisTrap FF) to separate the MHT-tag from PTPN14. PTPN14 was concentrated using a 15 kDa molecular weight cutoff (MWCO) Amicon centrifugal filter (Millipore) then size exclusion chromatography (SEC) on Superdex 200 (Cytiva, SD200) was used as the final purification step. The final concentration of PTPN14 was determined by measuring absorbance at 280nm and PTPN14 was stored in 20 mM HEPES, pH 7.5, 150 mM NaCl, 1 mM TCEP at −80°C for all subsequent experiments.

### Biolayer Interferometry

BLI assays were performed using the Gator^™^ Label-Free Bioanalysis instrument using Strep-Tactin-XT (ST-XT) sensor probes (Gator Bio, Palo Alto, CA, USA). The ST-XT probes were coated with purified twin-strep tagged PTPN14 phosphatase domain. A binding shift was detected after incubating the PTPN14-coated probes with different concentrations of purified E7 proteins. Buffer alone was used as reference, and MBP protein alone was used as a negative control. The dissociation constant for each protein pair was determined using the Gator One 2.7 software version 2.15.5.1221.

## Supplementary Material

Supplement 1

Supplement 2

Supplement 3

## Significance

Inactivation of the retinoblastoma tumor suppressor (RB1) is necessary but insufficient for HPV E7-mediated immortalization of human cells. In addition to inactivating RB1, HPV E7 proteins also target for degradation PTPN14, a tumor suppressor and inhibitor of the YAP1 oncoprotein. We report genetic complementation experiments demonstrating that RB1 inactivation and PTPN14 inactivation are separate activities of E7. Either depletion of RB1 or PTPN14 can confer lifespan extension activity on a low-risk HPV E7 and reduced levels of either tumor suppressor increases the expression of certain cell cycle genes. These findings redefine our understanding of the transforming activity of the E7 oncoprotein. Inactivation of two tumor suppressors is required for E7 activity and our findings support that targeting either E7/RB1 or E7/PTPN14 would be of therapeutic benefit. We propose that synergistic control of cell cycle gene expression by E2F and YAP1-dependent transcription is essential for the transforming activity of oncogenic HPV.
