# Supplementary material for: Inactivation of the RB1 and PTPN14 tumor suppressors cooperatively enables the carcinogenic activity of the human papillomavirus E7 oncoprotein
Source: bioRxiv. 2026 Mar 17:2026.03.16.712171. Preprint. [Version 1] doi: 10.64898/2026.03.16.712171 (PMC13015474; doi:10.64898/2026.03.16.712171)

bioRxiv preprint doi: https://doi.org/10.64898/2026.03.16.712171; this version posted March 17, 2026. The copyright holder for this preprint (which was not certified by peer review) is the author/funder, who has granted bioRxiv a license to display the preprint in perpetuity. It is made available under aCC-BY 4.0 International license.

Supplemental Figure 1: Characterization of HPV18 E7 mutants. Primary human keratinocytes were transduced with retroviral vectors encoding GFP, wild-type HPV18 E7 (WT), HPV18 E7 R84S, HPV18 E7 ∆DLLC, or HPV18 E7 R84A L91A. Retroviral vectors encoded neomycin or hygromycin resistance markers, as indicated. Each version of HPV E7 has an HA epitope tag at its C-terminus. (A) Total cell lysates were subjected to western blotting and probed with antibodies to PTPN14, GFP, HA, and actin. (B) The expression of E2F target genes (PCNA, CCNE2) was measured by qRT-PCR. (C) Total cell lysates were subjected to western blotting and probed with antibodies to PTPN14, RB1, GFP, HA, and actin. The expression of (D) E2F targets (PCNA, CCNE2) and (E) differentiation markers (KRT1, KRT10) was measured by qRT-PCR. (F) Cell populations were cultured for up to 51 days. Cells were counted at each passage and cell count data was used to calculate cumulative population doublings. In (D, E) bar graphs represent the mean ± range of two independent cell populations. Statistical significance was determined by one-way ANOVA followed by Holm-Sidak multiple comparison test, comparing each condition to the GFP control (*=$p \leq 0.05$; **=$p \leq 0.01$; ***=$p \leq 0.001$). In (B, F) data points represent individual measurements from a single experiment.

Supplemental Figure 2: Characterization of HPV16 E7 mutants in gene expression and lifespan extension assays. (A) Primary human keratinocytes were transduced with retroviral vectors encoding GFP, wild-type HPV16 E7 (WT), HPV16 E7 ∆DLYC, or HPV16 E7 R77S. All vectors carried a neomycin resistance marker. The expression of E2F target genes (PCNA, CCNE2) and differentiation markers (KRT1, KRT10) was measured by qRT-PCR. Bar graphs represent the mean ± range of two replicate cell populations. Statistical significance was determined by one-way ANOVA followed by Holm-Sidak multiple comparison test, comparing each condition to the GFP control (*=$p \leq 0.05$; **=$p \leq 0.01$). (B) Cell populations from (A) were

bioRxiv preprint doi: https://doi.org/10.64898/2026.03.16.712171; this version posted March 17, 2026. The copyright holder for this preprint (which was not certified by peer review) is the author/funder, who has granted bioRxiv a license to display the preprint in perpetuity. It is made available under aCC-BY 4.0 International license.

cultured for up to 67 days. Cells were counted at each passage and cell count data was used to calculate cumulative population doublings. Data points indicate the mean and error bars indicate the range for two replicate cell populations. (C) Primary human keratinocytes were transduced with retroviral vectors encoding GFP, wild-type HPV16 E7 (WT), HPV16 E7 ΔDLYC, or HPV16 E7 R77S. All vectors carried a hygromycin resistance marker. The expression of E2F target genes (PCNA, CCNE2) and differentiation markers (KRT1, KRT10) was measured by qRT-PCR. Bar graphs represent the mean ± range of two replicate cell populations. Statistical significance was determined by one-way ANOVA followed by Holm-Sidak multiple comparison test, comparing each condition to the GFP control (*=$p \leq 0.05$; **=$p \leq 0.01$; ***=$p \leq 0.001$). (D) Cell populations from (C) were cultured for up to 67 days. Cells were counted at each passage and cell count data was used to calculate cumulative population doublings. Data points indicate the mean and error bars indicate the range for two replicate cell populations.

Supplemental Figure 3: HPV16 E7 mutant proteins that cannot inactivate RB1 or PTPN14 can complement each other *in trans*. Primary human keratinocytes were transduced with pairs of retroviruses encoding GFP, wild-type HPV16 E7 (WT), HPV16 E7 ΔDLYC, or HPV16 E7 R77S, with one hygromycin-resistant and one neomycin-resistant retrovirus included in each condition. Each version of HPV16 E7 has an HA epitope tag at its C-terminus. (A) Total cell lysates were subjected to western blotting and probed with antibodies to PTPN14, RB1, GFP, HA, and actin. The expression of (B) E2F target genes (PCNA, CCNE2) and (C) differentiation markers (KRT1, KRT10) were measured by qRT-PCR. Bar graphs represent the mean ± range of two replicate cell populations. Statistical significance was determined by one-way ANOVA followed by the Holm-Sidak multiple comparison test, comparing each condition to the GFP control (*=$p \leq 0.05$; **=$p \leq 0.01$).

bioRxiv preprint doi: https://doi.org/10.64898/2026.03.16.712171; this version posted March 17, 2026. The copyright holder for this preprint (which was not certified by peer review) is the author/funder, who has granted bioRxiv a license to display the preprint in perpetuity. It is made available under aCC-BY 4.0 International license.

Supplemental Figure 4. RNA-seq analysis of HFK expressing HPV16 E7 or HPV6 E7. Primary HFK were transduced with MSCV retroviral vectors encoding untagged HPV16 or HPV6 E7 proteins or GFP. (A) The expression of 16E7, 6E7, and PCNA was measured by qRT-PCR. Bar graphs represent the mean ± SD from three replicate cell pools. Statistical significance was determined by one-way ANOVA followed by Holm-Sidak multiple comparison test, comparing each condition to the GFP control (*=$p \leq 0.05$; **=$p \leq 0.01$). Following RNA-seq, GO enrichment analysis was conducted on the genes that were upregulated (≥1.5 fold higher expression and adjusted *p*-value ≤ 0.05) or downregulated (≥1.5 fold lower expression and adjusted *p*-value ≤ 0.05) in (B) HPV16 E7 cells relative to GFP cells or (C) HPV6 E7 cells relative to GFP cells. Selected pathways are indicated.

Supplemental Figure 5. RNA-seq analysis of HFK expressing HPV16 E7 or HPV6 E7 compared to the corresponding LxCxE mutant. (A) Primary HFKs were transduced with retroviral vectors encoding untagged HPV16 E7 or HPV16 E7 ΔDLYC proteins. PolyA selected RNA from HFK expressing HPV16 E7 or HPV16 E7 ΔDLYC was analyzed by RNA-seq. The volcano plot shows the gene expression changes in HFK HPV16 E7 vs HPV16 E7 ΔDLYC. Over 700 genes are differentially expressed with ≥ 1.5 fold change and adjusted *p*-value ≤ 0.05. 47.5% of the hallmark E2F target genes are differentially expressed in HFK HPV16 E7 vs HPV16 E7 ΔDLYC. (B) Primary HFKs were transduced with retroviral vectors encoding untagged HPV6 or HPV6 E7 ΔGLHC proteins. PolyA selected RNA from HFK expressing HPV6 or HPV6 E7 ΔGLHC was analyzed by RNA-seq. The volcano plot shows the gene expression changes in HFK HPV6 E7 vs HPV6 E7 ΔGLHC. Three genes are differentially expressed with ≥ 1.5-fold change and adjusted *p*-value ≤ 0.05. Selected E2F target genes are labeled.

bioRxiv preprint doi: https://doi.org/10.64898/2026.03.16.712171; this version posted March 17, 2026. The copyright holder for this preprint (which was not certified by peer review) is the author/funder, who has granted bioRxiv a license to display the preprint in perpetuity. It is made available under aCC-BY 4.0 International license.

Supplemental Figure 6. Purification of HPV E7 proteins. Recombinant MHT-E7 proteins were purified from bacteria. (A) Size exclusion chromatogram (SEC) profiles of the different E7 proteins used for affinity measurements. Fractions between the dotted lines were pooled and used for downstream BLI assays. (B) SDS-PAGE of pooled fractions for each MHT-E7.

Supplemental Figure 7: E7 expression and CRISPRi mediated PTPN14 knockdown in long-term complementation experiment. Primary human keratinocytes were transduced with a retrovirus encoding GFP, HPV16 E7, or HPV6 E7, plus a CRISPRi lentivirus and selected with puromycin and neomycin. KRT10 RNA levels were measured by qRT-PCR. Bar graphs represent the mean ± range of two replicate cell populations. Statistical significance was determined by one-way ANOVA followed by Holm-Sidak multiple comparison test, comparing each sample to GFP + sgNT (*=$p \leq 0.05$; **=$p \leq 0.01$).

Supplemental Figure 8: E7 expression and CRISPRi mediated PTPN14 knockdown in primary keratinocytes. Primary human keratinocytes were transduced with a retrovirus encoding GFP, HPV16 E7, HPV18 E7 or HPV6 E7 plus a CRISPRi lentivirus and selected with puromycin and neomycin. (A) PTPN14 RNA levels were measured by qRT-PCR. Bar graphs represent the mean ± range of two replicate cell populations. Statistical significance was determined by one-way ANOVA followed by Holm-Sidak multiple comparison test. Within a set of samples transduced with the same E7, each sgPTPN14 sample was compared to the corresponding sgNT sample (**=$p \leq 0.01$; ***=$p \leq 0.001$; ****=$p \leq 0.0001$). (B) Total cell lysates were subjected to western blotting and probed with antibodies to PTPN14, RB1, GFP, HA and actin. (C) KRT10 RNA levels were measured by qRT-PCR. Bar graphs represent the mean ± range of two replicate cell populations. Statistical significance was determined by one-way ANOVA followed by Holm-Sidak multiple comparison test. Within a set of samples transduced

bioRxiv preprint doi: https://doi.org/10.64898/2026.03.16.712171; this version posted March 17, 2026. The copyright holder for this preprint (which was not certified by peer review) is the author/funder, who has granted bioRxiv a license to display the preprint in perpetuity. It is made available under aCC-BY 4.0 International license.

with the same E7, each sgPTPN14 sample was compared to the corresponding sgNT sample (***=$p \leq 0.001$).

bioRxiv preprint doi: https://doi.org/10.64898/2026.03.16.712171; this version posted March 17, 2026. The copyright holder for this preprint (which was not certified by peer review) is the author/funder, who has granted bioRxiv a license to display the preprint in perpetuity. It is made available under aCC-BY 4.0 International license.

Supplemental Figure 1

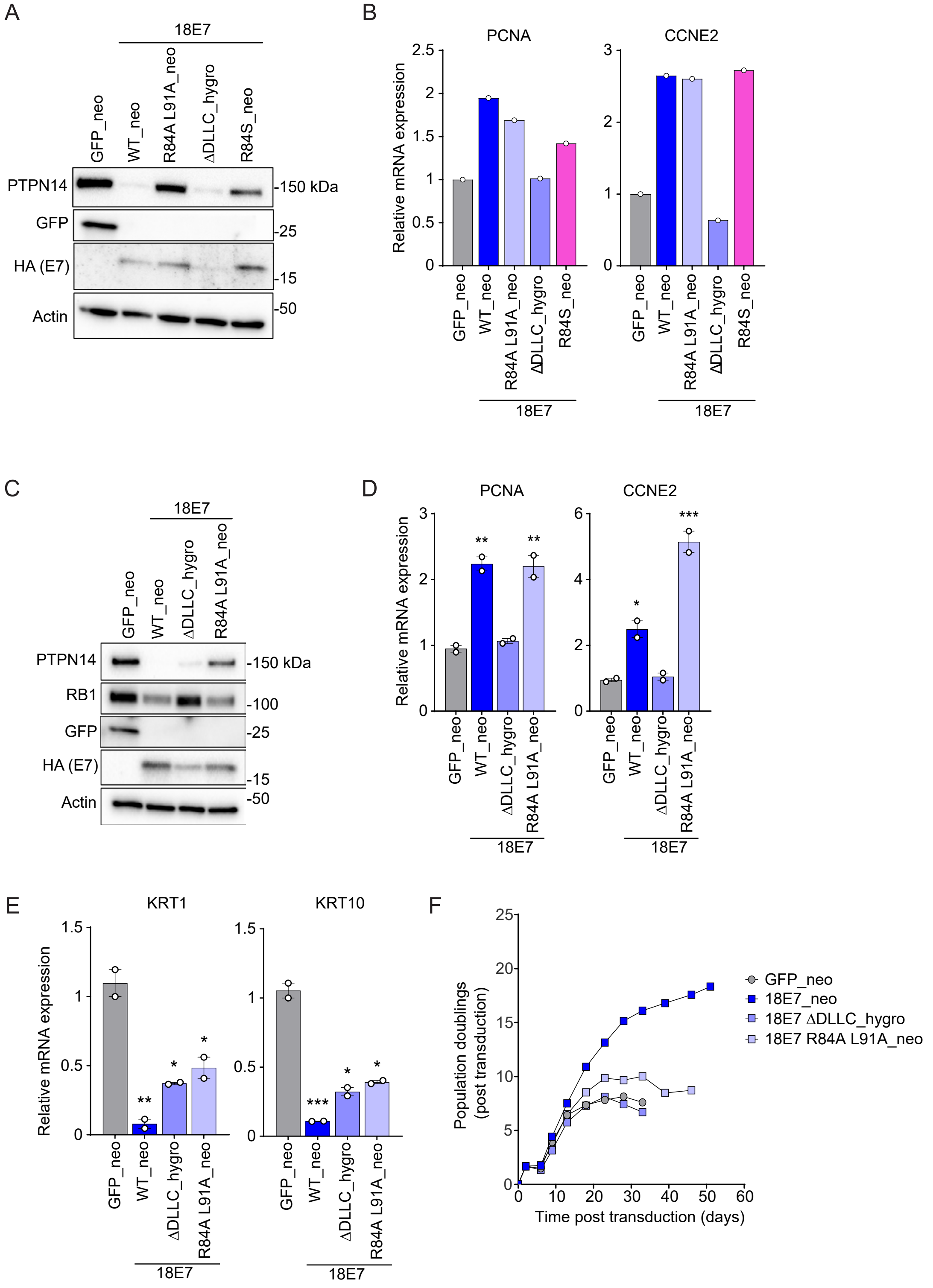

Supplemental Figure 2

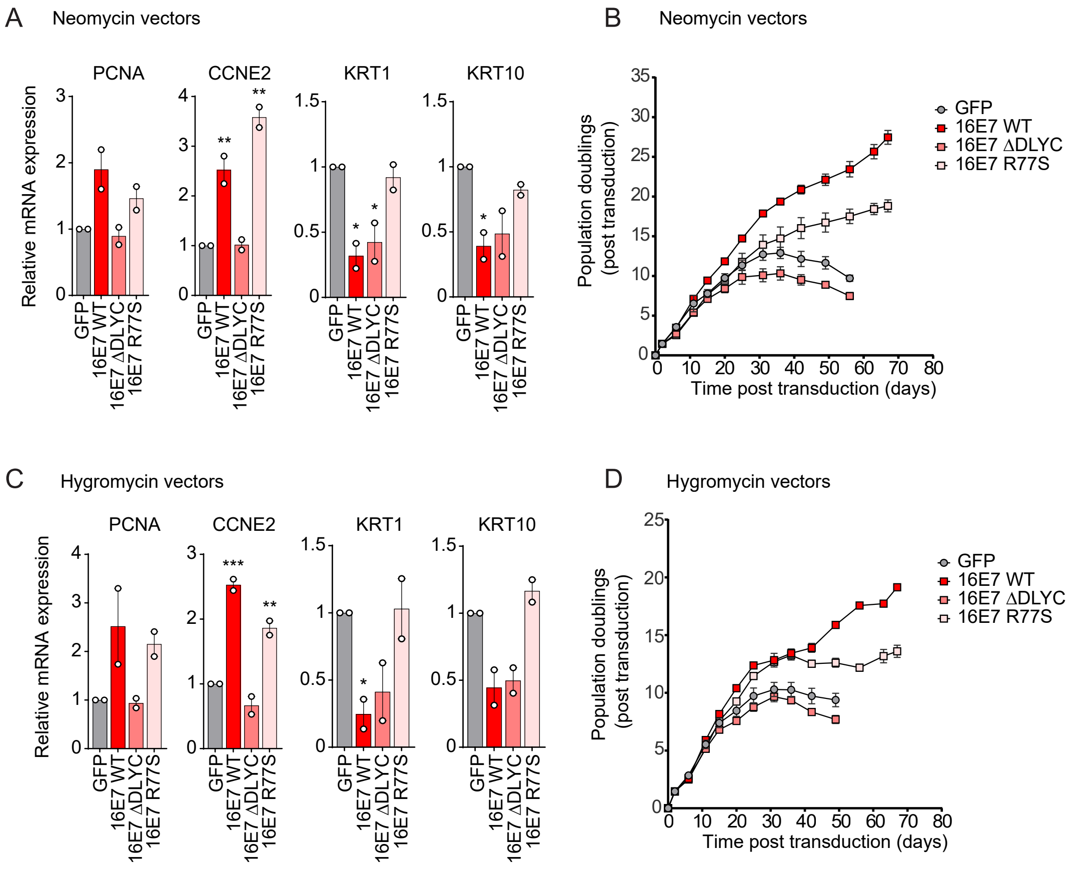

Supplemental Figure 3

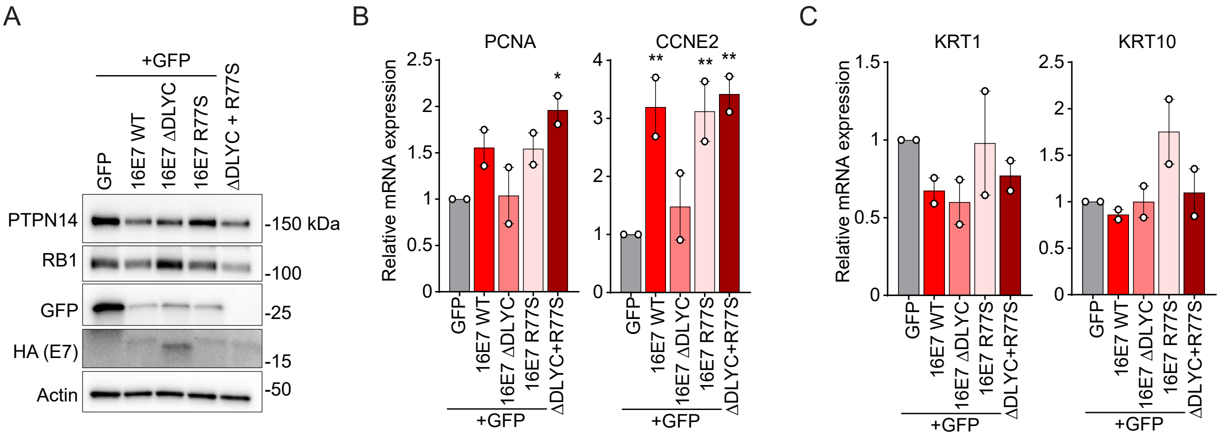

Supplemental Figure 4

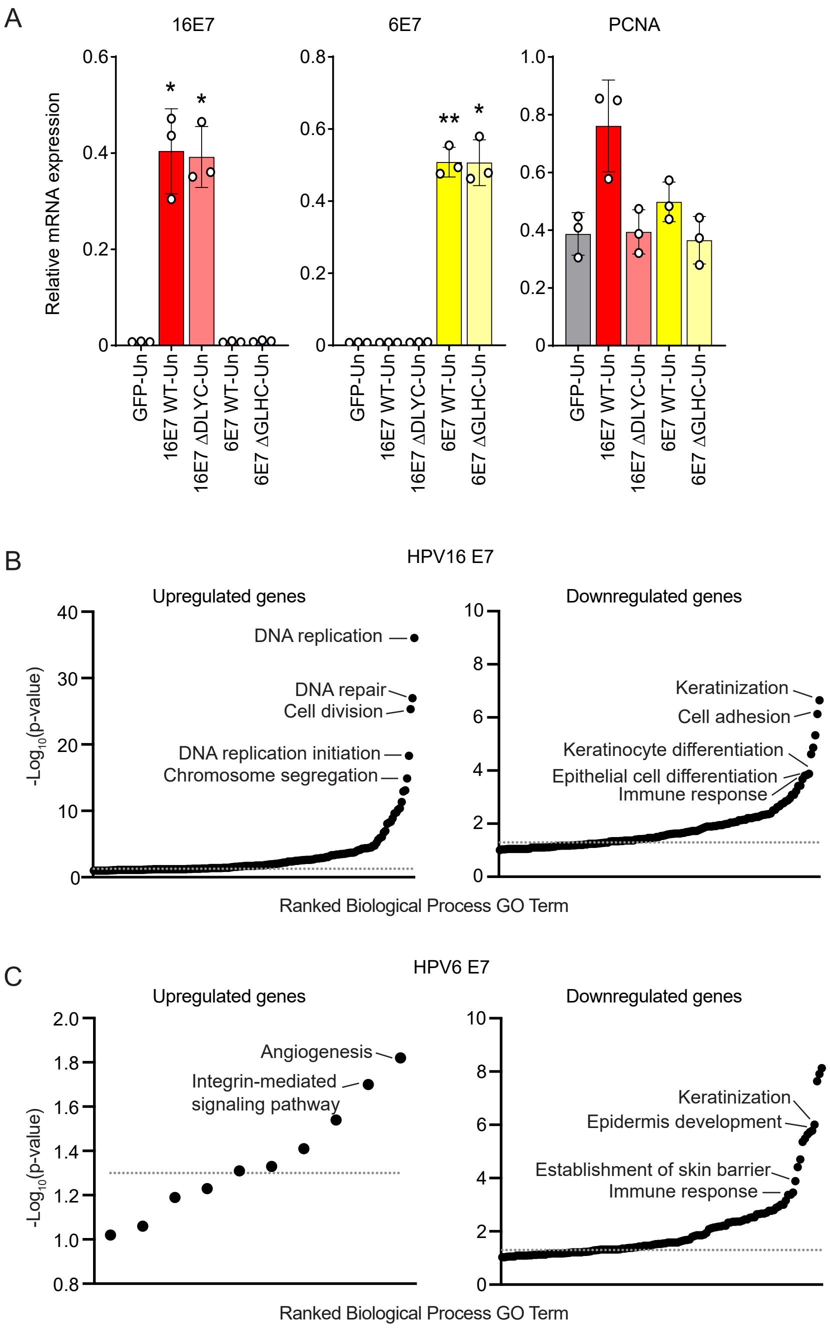

Supplemental Figure 5

A

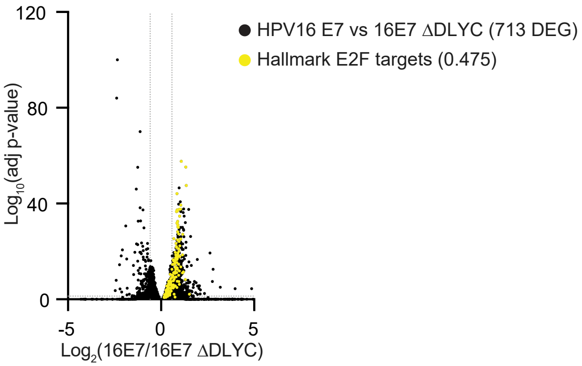

B

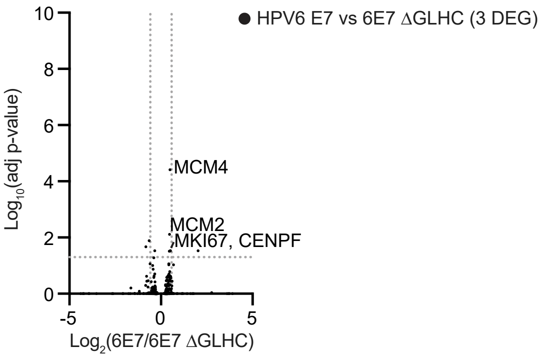

Supplemental Figure 6

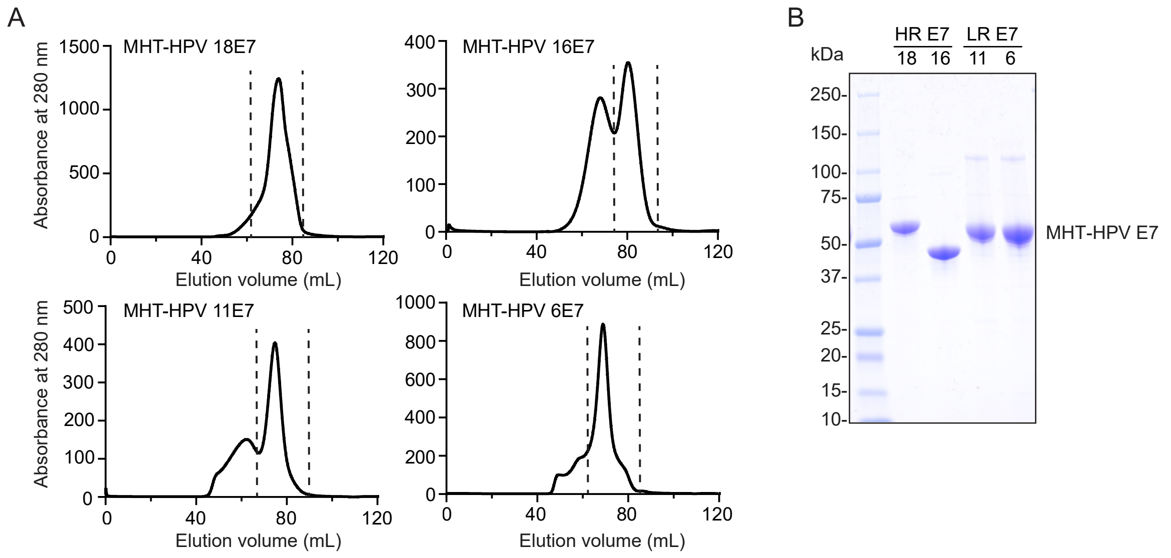

Supplemental Figure 7

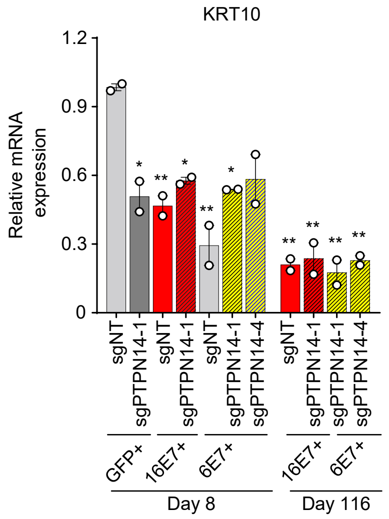

Supplemental Figure 8

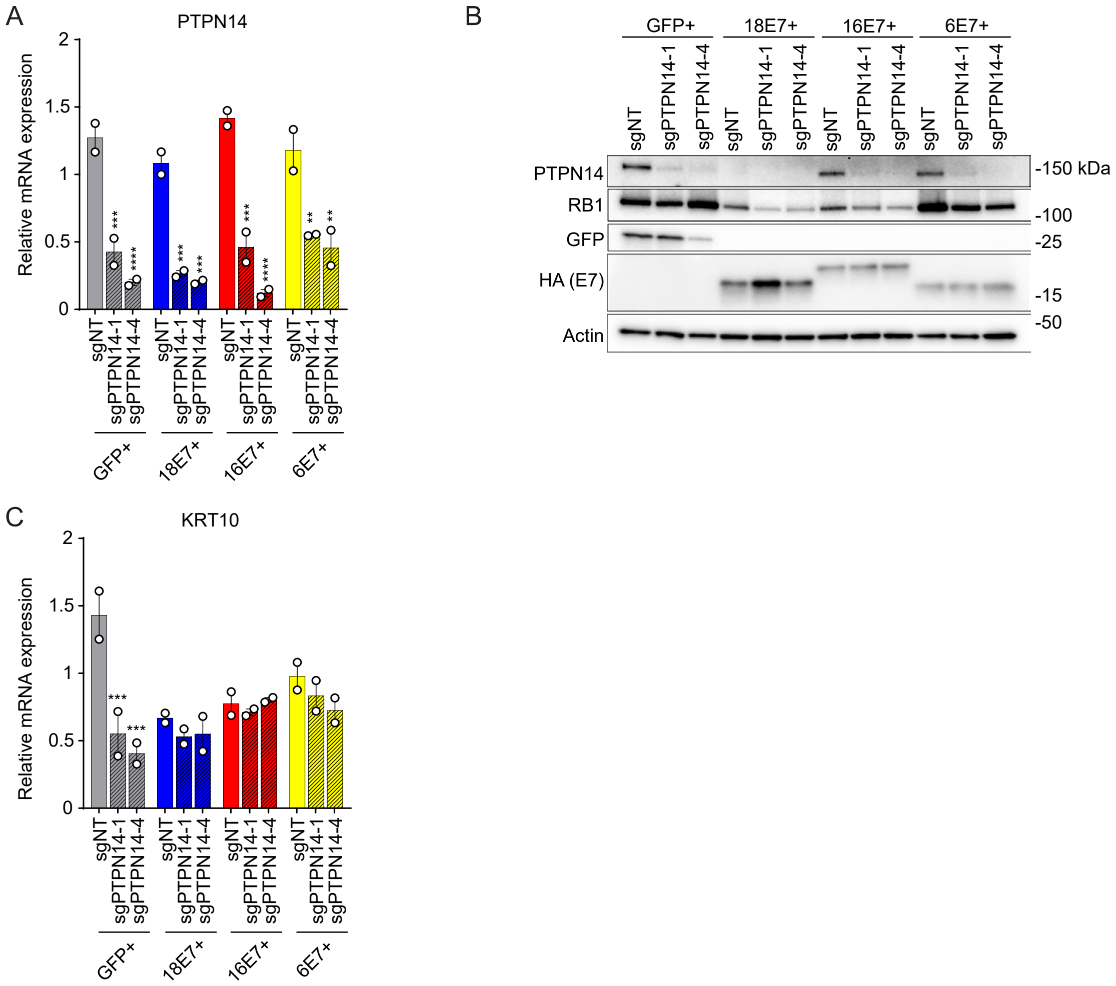

Supplement: Supplement 3 [file NIHPP2026.03.16.712171v1-supplement-3.pdf]
